# Anti-PD1-/PDL1-induced chronic intestinal pseudo-obstruction: three cases treated with vedolizumab after corticosteroid failure with mixed results

**DOI:** 10.1007/s00262-024-03901-4

**Published:** 2025-01-03

**Authors:** Morgan Zenatri, Michael Collins, Tifanie Alberto, Antonio Farina, Sophie Collardeau-Frachon, Mélanie Saint-Jean, François Bocquet, Frederic Dumont, Jérôme Honnorat, Bastien Joubert, Judith Raimbourg

**Affiliations:** 1 Department of Medical Oncology, Institut de Cancérologie de L’Ouest, 44805 Saint Herblain, France; 2 Nantes Université, Univ Angers, INSERM, CNRS, CRCI2NA, 44000 Nantes, France; 3 Institut Des Maladies de L’Appareil Digestif (IMAD), Hépato-Gastro-Entérologie et Assistance Nutritionnelle, Inserm CIC 1413, Inserm UMR 1235, Nantes Université, CHU Nantes, 44000 Nantes, France; 4 Department of Neurology, CRC SEP, Centre Hospitalier of Lille, Lille, France; 5 French Reference Centre on Paraneoplastic Neurological Syndromes and Autoimmune Encephalitis, Hospices Civils de Lyon, Hôpital Neurologique, 69677 Bron, France; 6 MeLiS-UCBL-CNRS UMR 5284, INSERM U1314, University Claude Bernard Lyon 1, 69008 Lyon, France; 7 Department of Pathology, Hospices Civils de Lyon & Université Claude Bernard Lyon 1, Lyon, France

**Keywords:** Chronic intestinal pseudo-obstruction, Anti-PD1, NSCLC, Vedolizumab

## Abstract

Immune checkpoint inhibitors (ICI), i.e., anti-PD1/PDL1 and anti-CTLA-4, have reshaped the prognosis of many cancers. Increased use of ICI has led to the onset of new adverse events. Neurological immune-related adverse events are rare, heterogenous, and potentially life-threatening. Chronic intestinal pseudo-obstruction (CIPO) is an immune-related autonomic plexus neuropathy that may be caused by infiltration of the myenteric plexus by CD8 + T cells. It is a rare and potentially fatal side effect that can be difficult to diagnose early because of initial nonspecific clinical presentation including vomiting, nausea, diarrhea, and constipation. Some rare cases have been described in the literature reporting a frequent resistance to corticosteroids making it necessary to use other immunosuppressive therapy. Vedolizumab is an antibody (Ab) blocking integrin α4-β7 used to treat inflammatory bowel disease. We report the first three cases of ICI-induced CIPO-treated with vedolizumab after corticosteroid failure, with very limited benefits (only one patient with transitory improvement). Based on our results in three cases, vedolizumab does not currently appear to be a therapeutic option. Earlier administration with a standardized dose and frequency schedule may provide better outcomes.

## Introduction

CIPO is a very rare and serious neurological side effect of IC (anti-PD1/PDL1/CTLA-4 Ab). It is a severe digestive syndrome characterized by derangement of gut propulsive motility which resembles mechanical obstruction, in the absence of any obstructive process [1]. CIPO physiopathology is not well understood but is possibly related to infiltration of the myenteric plexus by CD8 + T cells. Definite diagnosis is based on myenteric plexus biopsy, which can be difficult to obtain. The diagnosis is therefore one of elimination, made after a complete work-up (CT scan/MRI, endoscopy, manometry, laboratory test), in search of obstructive process, confirm impairment of gastrointestinal motility, and rule out a secondary cause of CIPO (infections, autoimmune disorders, degenerative neuropathies, mitochondrial disorders) [2, 3]. Due to frequent clinical degradation, it is necessary to evoke CIPO early, which can be challenging, because of initially nonspecific digestive symptoms. CIPO must be discussed when facing a patient treated with ICI with occlusive syndrome and no mechanical obstruction. Six cases have been published in the literature so far, with an inconstant response to corticosteroids. No other standard immunosuppressive therapy is validated. Vedolizumab an Ab specifically targets α4β7 integrin, involved in recruiting T lymphocytes to the intestinal wall [4, 5]. We report the first three cases of ICI-induced CIPO treated with vedolizumab.

## Description of the three cases

### Case 1

A 48-year-old woman, with a history of heavy smoking, was diagnosed in November 2020 with stage IVb poorly differentiated non-small cell lung cancer (PDL1 30%) with multiple metastasis sites (bone, brain, and lymph node).

Treatment with pembrolizumab, cisplatin, and pemetrexed started in December 2020. Since the first cycle, the patient presented progressively worsening constipation and became resistant to prokinetics. After the fourth cycle (February 2021), the patient was hospitalized because of severe constipation, daily postprandial vomiting, and weight loss.

**Fig. 1:**
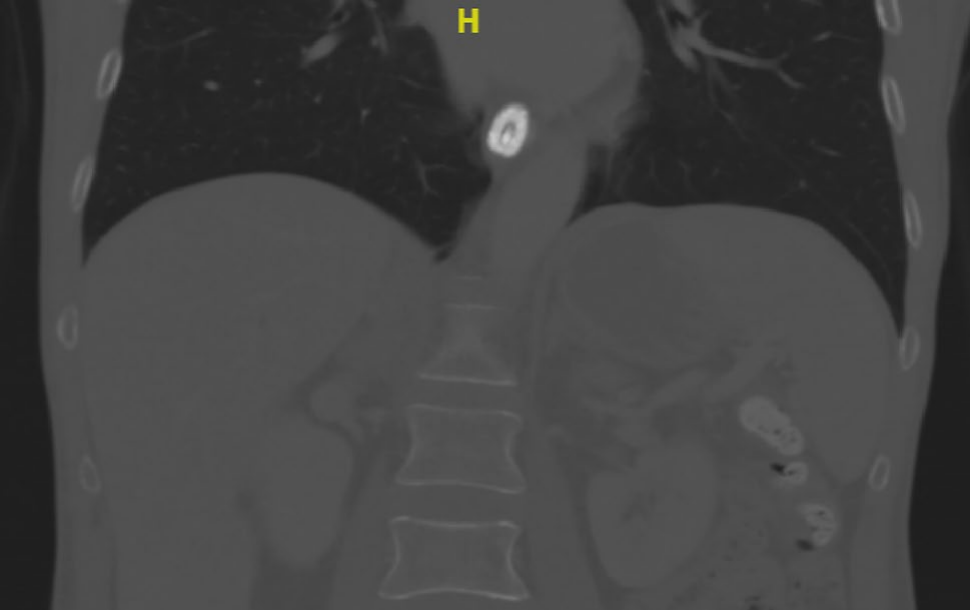
Coronal CT scan from patient 1 performed approximately 24 h after capsule endoscopy procedure showing the capsule stuck in the lower third of the esophagus

Abdominal CT scan showed no mechanical occlusion and suggested paralytic ileus. Upper and lower digestive tract endoscopy showed no mucosal lesions, and multiple biopsies were collected. Capsule endoscopy got stuck in the lower third of the esophagus (Fig. 1). Whole-body FDG-PET and brain MRI showed a complete response to cancer treatment. Cerebrospinal fluid (CSF) analysis and spine MRI (looking for spinal cord compression) were normal; neural Ab testing (serum and CSF) was negative.

The diagnosis of ICI-related intestinal pseudo-occlusion was suspected. Paraneoplastic syndrome (PNS) Ab was not detected, but this does not rule out a PNS, which may occur without PNS Ab and be triggered or worsened by ICI [6]. Pembrolizumab was definitively stopped; intravenous methylprednisolone 120 mg daily, prokinetics, and parenteral nutrition were started.

**Fig. 2:**
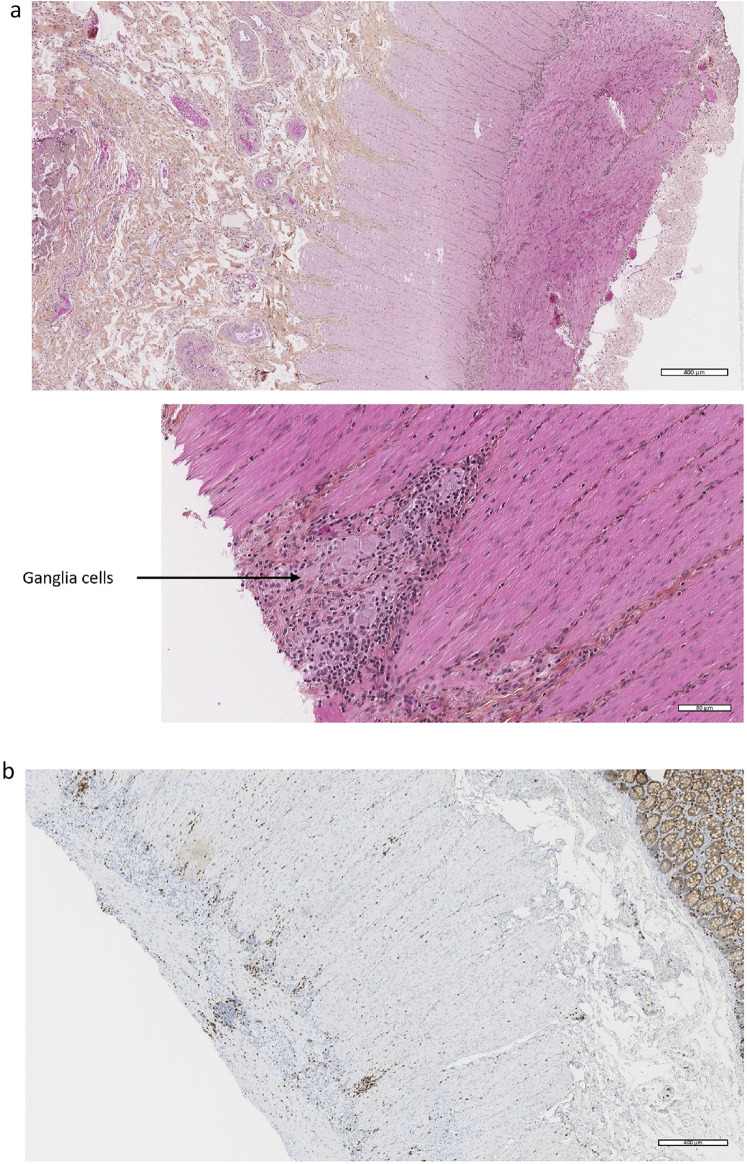
Intestinal biopsies from patient 1. **a** Inflammatory lymphocytic infiltrate around ganglia cells in myenteric plexuses of the muscularis (hematoxylin and eosin staining). **b** Immunohistochemistry: CD8 + T inflammatory infiltrate around and inside myenteric plexus of the muscularis

The diagnosis of immune-related enteric neuropathy was confirmed by the results of the intestinal biopsies (Fig. 2), showing lymphocytic, CD8 + predominant infiltrates in the myenteric plexus. Diagnostic work-up was then completed by esophageal manometry (test analyzing esophageal pressures showing type 3 achalasia) and nociceptive laser-evoked potentials (test looking for damage to small myelinated type A delta or unmyelinated type C fibers finding in our case an alteration of A delta fiber transmission from foot stimulation and demonstrating the presence of a small fiber neuropathy). Conversely, SUDOSCAN (test stimulating sudorific glands and assessing the function of small C-type nerve fibers) and the sympathetic skin reflex were normal.

One month later, corticosteroids and prokinetic had failed to improve symptoms. Therapeutic plasma exchange (TPE) was started; after the first TPE cycle, blood pembrolizumab levels decreased (from 22 to 7.5 mg/L), and a transient clinical improvement was observed (intestinal transit recovery, resumption of partial oral feeding). No further improvement was observed after a second TPE cycle, even though pembrolizumab levels further decreased (from 7.5 to 3.5 mg/L).

Escalation with vedolizumab (300 mg, 1 cycle every 8 weeks) was decided. During the 5 cycles of this treatment (May 2021–September 2021), the patient’s clinical status fluctuated: alternation of exclusive parenteral feeding and partial oral feeding, transient recovery of intestinal transit with alternate bowel occlusion. In addition, she had (as a complication of her prolonged fasting) acute lithiasic pancreatitis that required cholecystectomy.

In September 2021, the patient was treated with pemetrexed because of multiple lymph node progression with no response after two cycles. Gemcitabine was interrupted after one cycle due to multiple infectious complications, and best supportive care was recorded. In March 2022, a permanent drainage gastrostomy was performed, and the patient required daily gastric emptying from then till her death.

### Case 2

A 61-year-old man with a history of diabetes mellitus and hypertension was diagnosed in July 2020 with a locally advanced lung adenocarcinoma PDL1: 0%. He received first-line carboplatin, pemetrexed, and pembrolizumab, followed by maintenance pembrolizumab from January 2021. The patient presented several immune-related grade 2 toxicities: thyroiditis, pruritus, and pituitary insufficiency. The best response achieved was a partial response maintained until September 2021. Isolated progression of the primitive lesion was treated with thoracic radiotherapy with a partial response.

In August 2021, after one year of anti-PD1 treatment, the patient complained of alternating diarrhea and constipation. One month later, he was hospitalized for an occlusive syndrome associated with acute urine retention. The thoraco-abdomino-pelvic CT scan showed only aerogrelia and a dolichocolon with no junction syndrome. The exploratory laparoscopy found a 1.6-cm mesenteric metastatic nodule that did not explain the occlusion. An upper digestive tract fibroscopy revealed simple gastritis. The biopsy showed congestive gastric mucosal antritis within normal limits. Whole-body FDG-PET showed no pathological hyperfixation at the subdiaphragmatic level and no sign of disease progression. Serum auto-Ab (Hu, Yo, RI, CV2, amphiphisin, MA1 and 2, LGI1 and CASPR2) testing was negative.

The diagnosis of ICI-induced CIPO was suspected. Treatment with pembrolizumab was definitively stopped. He received prostigmine and metoclopramide without any efficacy. Intravenous corticosteroids at 2mg/kg were used during 2 weeks without improvement in clinical symptoms. The patient required exclusively parenteral nutrition, a discharge gastrostomy, and bladder self-catheterization.

Because of corticosteroid failure, vedolizumab (4 injections at 300mg (W0, W2, W6, W10)) was started in February 2022 with no improvement in the CIPO. Vedolizumab was permanently discontinued in September 2022. Infliximab was not chosen because of the infectious risks intrinsic to CIPO and parenteral nutrition, and TPE was not considered because of logistical problems.

No anti-neoplastic therapy was resumed despite metastatic progression occurring in January 2022, because of iterative sepsis and persistent CIPO, leading to the patient’s death in September 2022.

### Case 3

A 40-year-old non-smoking woman was diagnosed with small cell lung carcinoma in September 2021, which was initially metastatic to lymph nodes, pleura, pelvis, and bone. She was treated with 4 cycles of carboplatin—etoposide—atezolizumab, followed by atezolizumab maintenance. She had a partial metabolic response on whole-body FDG-PET scan.

In December 2021(3 months after initiation of atezolizumab), she developed very severe constipation with postprandial nausea and vomiting unrelieved by laxatives. In February 2022, nystagmus appeared.

**Fig. 3:**
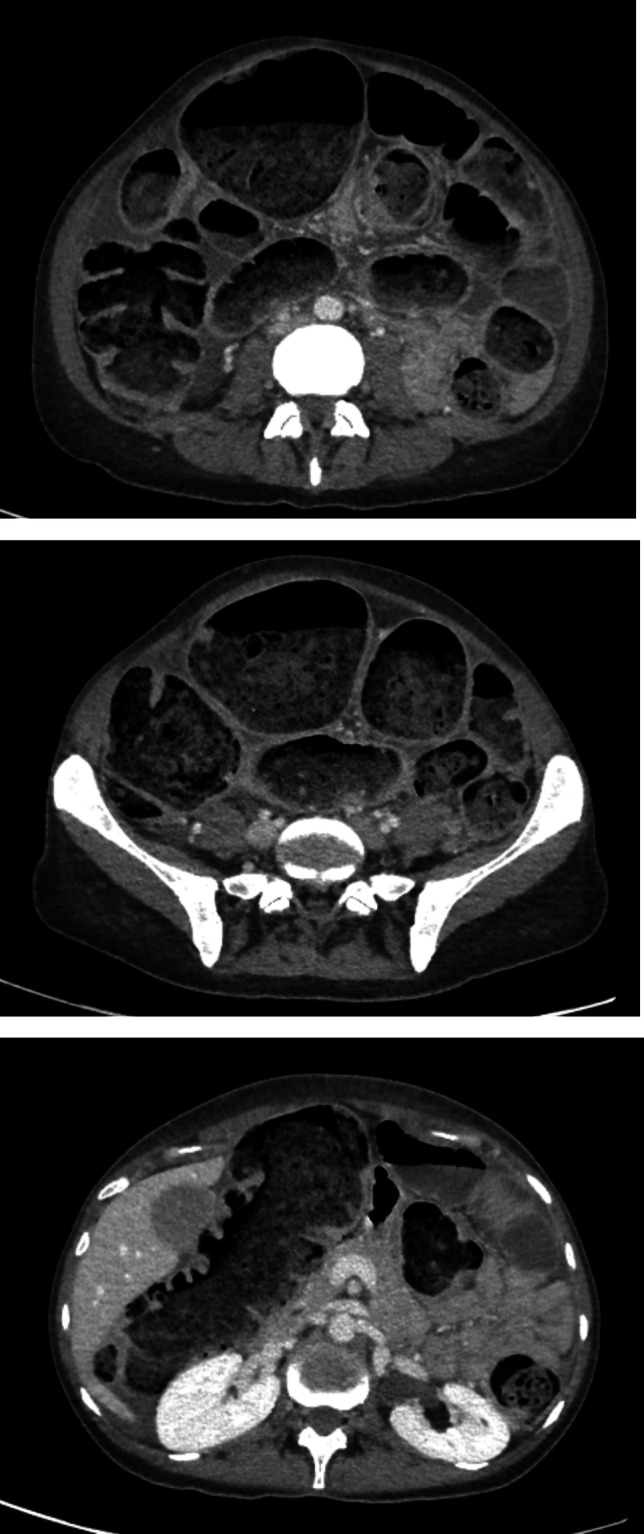
CT scan from patient 3 revealed a pseudo-occlusive syndrome

Brain MRI was normal. The thoraco-abdomino-pelvic CT scan revealed a pseudo-occlusive syndrome with no mechanical occlusion, attributed to a functional origin (Fig. 3). There were no other clinical signs of dysautonomia. Serum and CSF were negative for anti-neuronal Ab. CSF was moderately inflammatory, predominantly lymphocytic, with oligoclonal band, IL-6 14 pg/mL, atypical fluorescence for anti-neuronal Ab in the CSF and serum. These elements suggest a dysimmune disorder with an as yet unidentified anti-neuronal antibody.

Electromyogram and SUDOSCAN were normal. Repeated FDG-PET scans confirmed the persistence of a partial metabolic response.

Treatment with atezolizumab was definitively stopped. High-dose corticosteroid (1g) therapy for five days IV then 6 weeks PO, and IV immunoglobulin therapy were introduced. Three months later, the patient described an improvement in her balance disorders. She was able to resume a normal diet.

**Fig. 4:**
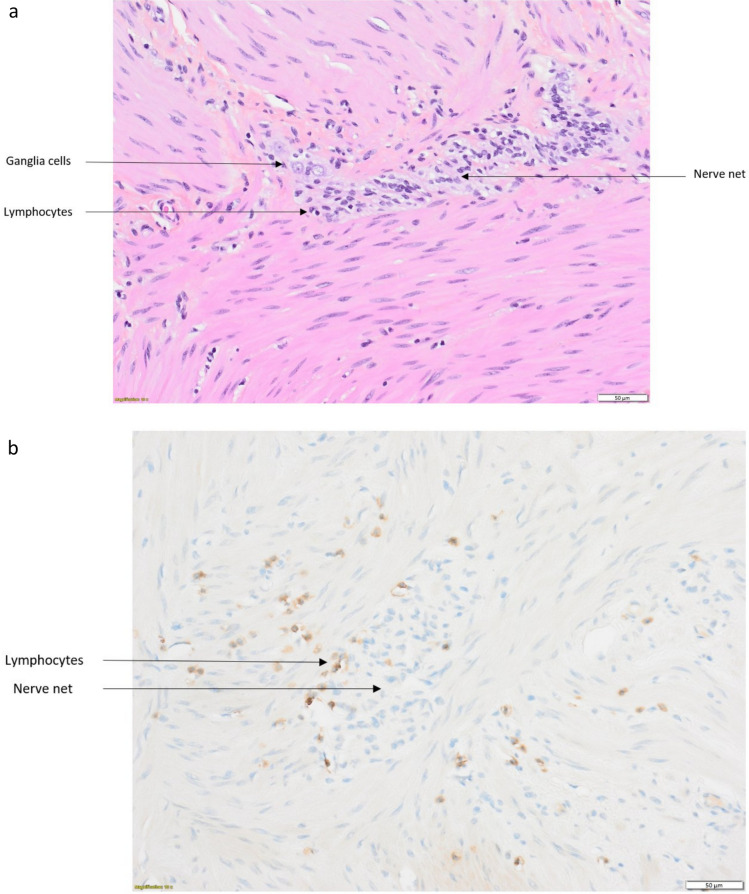
Intestinal biopsies from patient 3. **a** Lymphocytes in contact with a nerve net and ganglia cells (H&E staining). **b** Immunohistochemistry: CD8 + T inflammatory infiltrate around a nerve net

Three months later, in September 2022, isolated multidirectional nystagmus without cerebellar ataxia and severe constipation resistant to laxatives relapsed. The presence of nystagmus could be explained by the presence of an overlap syndrome between central nervous system damage responsible for nystagmus and autonomic nervous system damage responsible for CIPO. A discharge colostomy was performed because of scannographic signs of digestive suffering. Esophago-duodenal fibroscopy and colonoscopy were normal. Colonic biopsies in June 2023 revealed a parietal inflammation, with a CD8 + T lymphoid population in contact with the nerve nets and ganglioneural cells of the digestive musculature (Fig. 4). The diagnosis of CIPO was retained. Transit was subsequently well regulated by the colostomy suggesting limited impairment to the colon. Treatment with vedolizumab (300 mg, W0 W2 W6) was introduced in July 2023 with no improvement to the nystagmus as yet. Treatment with carboplatin and etoposide was introduced instead of atezolizumab.

To date, the patient is alive with no evidence of disease; digestive continuity has not been restored. The intention is to perform a colonic biopsy, and in the event of a significant reduction in the inflammatory infiltrate, to try to restore digestive continuity.

## Discussion

We report three cases of anti-PD1-/PDL1-induced CIPO resistant to corticosteroids that were treated with vedolizumab. Only six cases of ICI-induced CIPO have been reported in the literature [7–12]. Five out of six were induced by anti-PD1/PDL1 treatment for the treatment of small cell lung cancer (n = 2, one patient treated with atezolizumab and one with nivolumab), lung adenocarcinoma (n = 1, with nivolumab), gastroesophageal junction adenocarcinoma (n = 1, with pembrolizumab) and poorly differentiated neuroendocrine carcinoma (n = 1, with pembrolizumab). The five patients were treated with corticosteroids with favorable evolution in three patients (recovering total food intake). The two others failed to respond to corticosteroids and one of them died quickly of septic shock. No other immunosuppressive treatment was used. Nivolumab was reintroduced in two patients who had improved after corticosteroids: one patient did not relapse, while the other presented the same digestive symptoms at D4, with renewed efficacy of corticosteroids. The last case report involved a patient treated with only one course of nivolumab and ipilimumab for Merkel cell carcinoma. The patient’s evolution was unfavorable despite corticosteroids, tacrolimus, and TPE. The patient died of septic shock due to digestive perforation.

CIPO occurs with pseudo-obstructive symptoms, without mechanical obstruction. Complementary investigations are often negative, and the diagnosis is confirmed by mesenteric biopsy though it is rarely performed. In routine practice, the diagnosis is made by exclusion.

The pathophysiology of ICI-induced CIPO is not fully understood. It may be induced by an infiltration of the myenteric plexus by CD8 + T cells. Paraneoplastic CIPO and immune-related paraneoplastic CIPO have been described, notably related to anti-Hu [13, 14] and anti-CV2/CRMP5 [15] Ab, but no PNS Ab were found in our three cases. Corticosteroid response is not consistent and there is no other standardized immunosuppressive therapy [16]. Infliximab and vedolizumab are used in inflammatory bowel disease and are use in the management of ICI-induced colitis. The use of these both drug in ICI-induced CIPO could be discuss. Infliximab inhibits the binding of the inflammatory cytokine TNFα to its receptors, reducing both local and systemic inflammation. Vedolizumab, a monoclonal Ab, inhibits the α4-β7 integrin present only in the gastrointestinal tract, which allows the passage of lymphocytes from the blood to the digestive mucosa. There is no effect beyond the digestive tract. Furthermore, in a meta-analysis comparing both drug in the treatment of inflammatory bowel disease, vedolizumab showed similar response rates to infliximab but with better safety outcomes and may be less immunosuppressive, which can be interesting for patients with cancer and an immune-related CIPO at very high risk of septic complication [17, 18]. The mechanism of action and safety profile of vedolizumab make it the preferred option. Extracorporeal photopheresis could be also discussed, with efficacy reported in cases of severe post-ICI-related colitis [19].

We report here the first three cases of patients treated with vedolizumab for a corticoresistant immune-related CIPO induced by anti-PD1/anti-PDL1 therapy. Results are weak, with only a mild transient benefit for one patient. The reasons for this low efficacy are not known, but hypotheses include delayed introduction with irreversible or extensive plexus damage. Another hypothesis is that the CIPO digestive damage may be more or less extensive, involving all or part of the digestive tract. This could explain various responses to corticosteroids too. Alternatively, the mechanism of action of vedolizumab is not adapted to neurological lesions induced by ICI.

This is a retrospective case study of only three cases which inevitably presents biases. The patients suffered from different types of cancer. Their clinical presentation differed, with digestive involvement associated, depending on the case, with urinary or central neurological impairment. The immunosuppressive agents used as first-line treatment were different, as were the timing and schedule of vedolizumab administration.

To conclude, CIPO is a rare and serious adverse effect of ICI that clinicians should be aware of, in cases of unexplained occlusive syndrome to avoid therapeutic delays. Corticosteroids are the first treatment of choice but frequent fail. For second-line immunosuppressive therapy, our data showed limited benefits of vedolizumab that could be due to a late introduction of vedolizumab. Our data do not support for the moment the use of vedolizumab. More research is needed to find the most appropriate treatment for this rare but potentially fatal adverse event.

## Data Availability

No datasets were generated or analyzed during the current study.
